# Characteristics and quality assessment of online mentoring profile texts in academic medical mentoring

**DOI:** 10.1186/s12909-023-04804-1

**Published:** 2023-11-09

**Authors:** Jonathan A. Gernert, Maximilian Warm, Lukas Salvermoser, Nils Krüger, Stephan Bethe, Lorenz Kocheise, Malte von Hake, Charlotte Meyer-Schwickerath, Tanja Graupe, Martin R. Fischer, Konstantinos Dimitriadis

**Affiliations:** 1 Institute of Medical Education, LMU University Hospital, Pettenkoferstr. 8a, Munich, 80336 Germany; 2 Institute for Clinical Neuroimmunology, LMU University Hospital, Munich, Germany; 3 Department of Radiology, LMU University Hospital, Munich, Germany; 4 Department I of Internal Medicine, Faculty of Medicine and University Hospital Cologne, University of Cologne, Cologne, Germany; 5 Department of Neurology, LMU University Hospital, Munich, Germany; 6 Institute for Stroke and Dementia Research (ISD), LMU University Hospital, Munich, Germany

**Keywords:** Medical mentoring, Online matching, Online mentoring profiles

## Abstract

**Background:**

Mentoring is important for a successful career in academic medicine. In online matching processes, profile texts are decisive for the mentor-selection. We aimed to qualitatively characterize mentoring-profile-texts, identify differences in form and content and thus elements that promote selection.

**Methods:**

In a mixed method study first, quality of texts in 150 selected mentoring profiles was evaluated (10-point Likert scale; 1 = insufficient to 10 = very good). Second, based on a thematic and content analysis approach of profile texts, categories and subcategories were defined. We compared the presence of the assigned categories between the 25% highest ranked profiles with the 25% lowest ranked ones. Finally, additional predefined categories (*hot topics*) were labelled on the selected texts and their impact on student evaluation was statistically examined.

**Results:**

Students rated the quality of texts with a mean of 5.89 ± 1.45. 5 main thematic categories, 21 categories and a total of 74 subcategories were identified. Ten subcategories were significantly associated with high- and four with low-rated profiles. The presence of three or more *hot topics* in texts significantly correlated with a positive evaluation.

**Conclusion:**

The introduced classification system helps to understand how mentoring profile texts are composed and which aspects are important for choosing a suited mentor.

**Supplementary Information:**

The online version contains supplementary material available at 10.1186/s12909-023-04804-1.

## Main

### Introduction

Mentoring can significantly contribute to a successful academic career in medicine [1–4]. Increased satisfaction, enhanced academic success, acquisition of clinical and research skills are some of the positive effects attributed to mentoring. Nevertheless, its´ impact very much depends on the quality of the mentoring relationship. Dysfunctional mentoring can lead to a low sense of self-esteem, high levels of dissatisfaction and therefore adversely affect academic development [5].

Thus, finding a suitable mentor has a major influence on a successful mentoring relationship. Although, finding a suitable mentor during formal or informal academic events is said to be the most promising way to initiate a successful mentoring relationship, identifying the desired mentor can be a challenge [6, 7]. Therefore, most structured mentoring programs have implemented different matching strategies [8]. Traditionally, mentors are assigned during personal consultations with mentoring-program staff, where proteges get presented with different options, often by looking into mentoring profiles [9, 10]. In recent years, mentoring programs increasingly use electronic data processing (EDP)-supported matching procedures that were found to be as effective as personal consultations [11, 12]. These are based on online mentoring profiles of potential mentors, that often include basic curriculum vitae information, a picture and a text composed by the mentor.

Although potential proteges were able to identify key information on mentoring profiles that matched their expectations and goals for entering a mentoring relationship, the most mentioned reason for choosing a mentor was “likeability” based on the online profile text composed by the mentor [13]. Nevertheless, mentoring profile texts in most EDP-supported matching procedures are not standardized. Text-length, text-quality and type of information disclosed show a high degree of variability. This might be an advantage since length, form and context might provide valuable information about some character trades of the potential mentor and thus inform decision. On the other hand, lack of standardization might mislead student due to lack of important information. To our knowledge evidence on the influence of online mentoring profile texts on the selection of a suitable mentor is lacking.

Goal of this study was to characterize online mentoring profile texts, identify elements that could discriminate against positively evaluated and therefore often selected mentoring profiles. We focus on mentoring dyads between medical students and physicians in different positions.

## Material & methods

### Study design

The Ethics Committee of the Medical Faculty of LMU Munich waived approval for the study. A multi-method approach was applied to answer the above-mentioned research objectives. Since 2008, the Medical Faculty of LMU Munich runs a successful, large-scale mentoring program that allows medical students to match a suitable mentor among more than 400 possible candidates [14]. The mentoring program is voluntary for mentors, persons with a medical degree can create a profile. Although, students are offered the option to get a personal consultation with mentoring-program staff, most (80–90%) prefer to use one of the two EDP-supported matching procedures available. The first allows students to use search terms and apply filters to all eligible (confirmed by the mentor and fully filled in) online mentoring profiles. The second option suggests the 10-best mentor-matches, based on an algorithm that uses data from both the mentors and the proteges profile [11]. Students can then choose among these 10 mentors by looking into their online profiles. Mentoring profiles in our program are composed of 3 parts: i) Basic information like name, medical specialty, and additional qualifications; ii) a free profile text created by the mentor iii) an optional profile photo. The profile texts are created independently by each mentor without any formal or content specifications and are not subject to a correction process.

In a first step, we selected 150 online mentoring profiles that fulfilled certain criteria mentioned within the next section (Supplement Table 1). In a next step, profiles were evaluated and categorized into “high quality profiles” and “low quality profiles” by 11 students. Profile-texts were then anonymously extracted and analyzed by means of qualitative text analysis. The newly defined, inductive categories (see below) as well as predefined categories (which are known to be important to medical students in a mentorship [13] were used to label all profile-texts and perform quantitative analysis.

### Profile selection and rating

One hundred fifty online mentoring-profiles were anonymously extracted from our database when fulfilling following criteria: First, each selected profile had to contain i) basic demographic data and ii) a profile-text which had to be at least 200 characters long.

Eleven medical students, who were not part of the study team, were randomly selected as evaluators: 8 women and 3 men were selected, the majority had a mentor, and all had completed the preclinical section of medical school. These students ranked all 150 mentoring profiles on a Likert scale ranging from 1 to 10 (1 = insufficient; 10 = very good) based on the following question: “How do you assess the quality of the profile with regard to the selection of a suitable mentor (regardless of your personal preferences)?”. One data set was excluded due to an unplausible rating pattern. The mean and standard deviation was calculated for each profile.

**Fig. 1:**
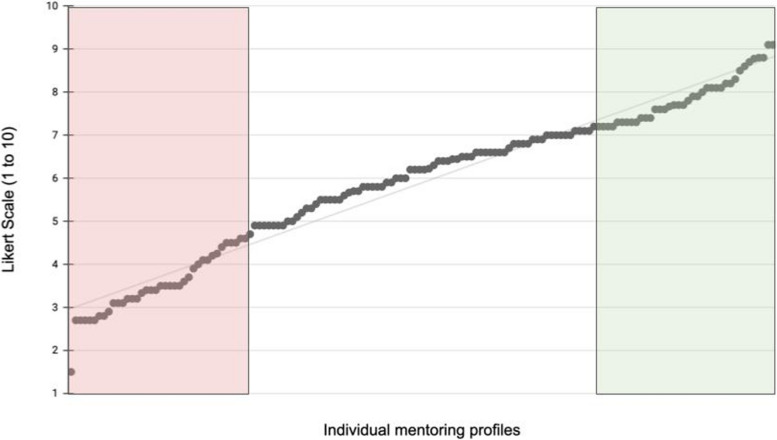
Mean evaluation of mentoring-profiles by students based on the question: “How do you assess the quality of the profile with regard to the selection of a suitable mentor (regardless of your personal preferences)?”. Mean rating of all 150 mentoring-profiles by 10 students on a 10-point Likert scale (1 = insufficient; 10 = very good). Quartiles with the low (red) and high (green) quality profiles were included for further analysis

All profiles were divided into quartiles and the 25% best rated and 25% worst rated profiles with a standard deviation less than 2 (for increased homogeneity among the selected profiles) were included in further analyses (Fig. 1). Thus, 38 “high quality profiles” and 35 “low quality profiles” were included to perform qualitative and quantitative text analysis.

### Data analysis

Two experienced researchers (LS, NK) independently defined categories using a thematic and content analysis approach. We used a modified approach to qualitative content analysis according to Kuckartz [15] and screened the profile texts for presence of new categories, until ten consecutive texts did not deliver any new items. Categories defined by the two raters were compiled. An agreement was reached between raters after discussing and resolving differences. These categories enabled us to systematically analyse the content and formal features of individual profiles in a standardized way.

Additionally, we used deductively predefined categories (which we named *hot topics)* based on a previous survey [13], that investigated medical students’ goals when entering a mentoring-relationship. The seven most frequently mentioned goals were defined as categories (these topics include information about “clinical electives”, “final year electives” and “research and MD thesis” as well as exchange about “personal goals”, “experience abroad”, “career planning” and “network and contacts”). Hypothesizing that availability of such content in a mentoring-profile text will increase its rating as a “high quality profile”, we coded for these additional categories.

Both sets of categories were used to analyse the selected online mentor-profiles by three blinded researchers (MW, LS, NK). All three researchers agreed on the anchoring examples. To avoid cognitive biases, calibrate responses and therefore increase reliability, coders were trained by using 20 mentoring-profiles. For the actual analysis each rater labelled the five major thematic categories with corresponding 74 subcategories and the seven main interest topics on every profile using a binary classification, depending on whether a category was present in the text or not.

### Statistics

Inter-rater reliability was measured using Cohen’s kappa. We applied a Chi-squared test to detect differences between “high quality profiles” and “low quality profiles” concerning the presence of the different categories and whether these profiles responded to the seven major interests of the students. A *p*-value < 0.05 was considered significant, the effect size was calculated using Cramer´s *V*. Microsoft Excel (Microsoft Corporation. (2018). Microsoft Excel, Redmond, Washington USA) and GraphPad Prism (version 8.0.0, GraphPad Software, San Diego, California USA) were used for statistical analysis and creation of the Figures.

## Results

### Mentoring profiles evaluation by students

Overall, students rated all 150 mentoring-profiles with 5.89 ± 1.45 (mean ± standard deviation). A linear distribution pattern was detected (Fig. 1). After exclusion of profiles (*n* = 3) with a standard deviation > 2 the upper (*n* = 38; 7.88 ± 0.58) and lower (*n* = 35; 3.42 ± 0.69) quartiles were further quantitatively assessed.

### Category system for mentoring profile text analysis

The qualitative analysis of all available profile texts yielded five main thematic categories, 21 categories and a total of 74 subcategories. A full list of all categories and the category-structure are presented in Supplement Table 2 and Supplement Figure 1.

The main thematic categories include 1) *formal* assessment of the text *e.g.*, salutation, structure, or additional references, 2) information on the mentor's *studies* and 3) *work* experience, 4) *mentoring* (*e.g.*, expectations of the mentor or his/her personal mentoring experiences) or 5) *personal* information, *e.g.*, on the mentor's family or hobbies.

As an example, for the main thematic category "studies", information about the doctoral thesis of the mentor was subsumed, which is divided into five further categories and the three subcategories "experimental research", "clinical research" and "statistical research" (Supplement Figure 1). The quote "I did my doctoral thesis in experimental nephrology in Munich." was used as an anchor example for the subcategory "experimental research". The exemplary quote “I did my doctoral thesis in haemato-oncology. The project was experimental and is completed.” from a profile text was therefore labelled as follows: "studies", "doctoral thesis" and the subcategory "experimental research".

### Quantitative mentoring-profile analysis

On average, the profiles contained 1,173 (range: 235 to 10,564) characters, the 38 highest rated profiles 1,385 (602 to 3,705) and the 35 lowest rated profiles 1,143 (238 to 10,564) (no statistically significant difference). Assessment of the 73 extracted mentoring-profiles according to the presence or absence of individual categories by three individual raters reached a Cohen's kappa of 0.8.

The most frequently represented subcategories were the statement "working at a university hospital" (category: “work”; 65/73), "working in patient care" (category: work; 61/73) and "fully formulated text" (category: formal; 49/73).

**Fig. 2:**
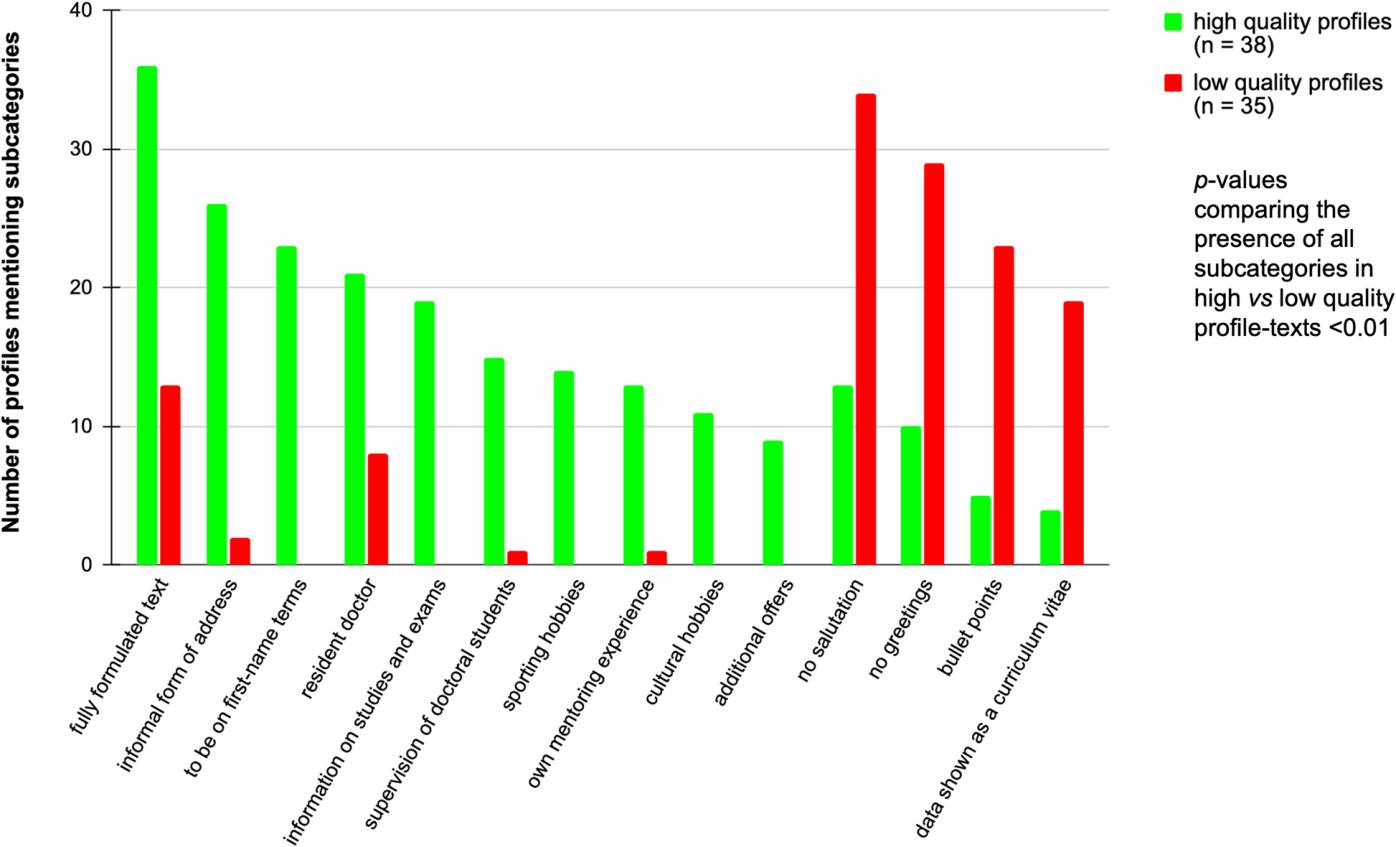
Subcategories with statistically significant correlation to student rating. Overall, ten categories show a significant correlation with high rated and 4 categories with low rated profiles by the students. The y-axis represents the frequency with which each subcategory is met. The significance levels of positively or negatively evaluated profiles according to their presence of each subcategory reached a *p*-value < 0.01 (Cramer´s V range 0.33 – 0.65, please also refer to Supplement Table 3)

We identified ten subcategories that significantly correlated with a higher student profile ranking (all with a *p*-value < 0.01; Fig. 2). Formal subcategories, including "fully formulated text", "informal form of address" and "to be on first-name terms" were all found in 23 to 36/38 high quality profile texts but only in 0 to 13/35 low quality profiles and seemed to have the most impact on student perception. Further subcategories with significant positive associations relate to "information on studies and exams", "supervision of doctoral students", "own mentoring experience", certain hobbies of the mentor or the mentor's current position as "resident doctor".

Further, four subcategories significantly correlate with low profile ranking (all with a *p*-value < 0.01; Fig. 2). All of them refer to the formal category of the free text: “no salutation”, “no greetings”, “bullet points” or “data shown as a curriculum vitae” (subcategories present in 19 to 35/35 low quality profiles but only 3 to 12/38 high quality profiles). The Cramer´s V ranges from 0.33 to 0.65, for detailed statistical analysis please refer to Supplement Table 3.

### Analysis of *hot topics* in medical mentoring

**Fig. 3:**
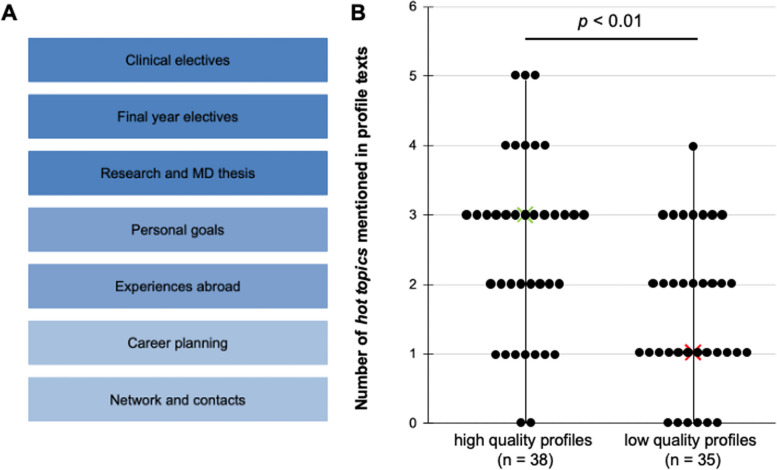
*Hot topics* in medical mentorship. **A**: Predefined *hot topics* in medical mentorship were used to analyse free text mentoring profiles [13]. **B**: High quality profiles (*n* = 38) address hot topics significantly more often (median: 3; interquartile range: 1) than low quality profiles (*n* = 35; median: 1; interquartile range: 1) (Qhi-squared test: *p*-value < 0.01, Cramer´s V = 0.33)

Results of the hot topics profile text analysis is presented in Fig. 3. The 38 high rated profile texts include information with a median of 3 (interquartile range: 1) *hot topics*. In contrast, half of the 35 low rated profiles deal with no or only one *hot topic* (median: 1; interquartile range: 1). Analysis of the profile texts also reveals that there is a significant association between the number of *hot topics* addressed and the student rating (Chi-squared test). Naming of at least three *hot topics* in profile texts correlates with a positive perception by the students (*p*-value < 0.01) (Fig. 3B).

## Discussion

By applying a mixed method analysis of online mentoring profile texts we could characterize content and form of texts and identify several elements that correlated with “high quality profiles”. We thereby identified 10 subcategories that significantly correlate with high profile ranking, respectively 4 subcategories with significantly low profile ranking by students. Further, the number of addressed *hot topics* shows a significant association with the student evaluation of the profile texts.

First, formal categories (*e.g.*, “fully formulated text", "informal form of address", "to be on first-name terms”, respectively, “no salutation”, “no greetings”, “bullet points”, “data shown as a curriculum vitae”) seem to impact students' perception with highest significance in both directions (positive respectively negative). In addition, there is a trend that positively rated profiles contain longer free text than negatively rated profiles. At first glance formal aspects do not seem to be related with likability, and therefore we interpret these findings as an indication that students use time and motivation of the mentor when creating a profile text as a decision criterion. However, the effort involved might give some hints about character traits of the mentor, which according to the Fundamental Principle of Liking (FPL) are again significantly influencing likeability [16]. In studies of likeability in the context of a court environment the notion of respectfulness is prominent [17]. Lack of effort in composing a profile text could be interpreted as disrespect and reduce likeability.

Second, the positive correlation of soft factors such as hobbies and other personal information with a positive profile rating reflect that student value an interpersonal connection when looking for a mentor. Personal aspects (hobbies) as well as mention of personal struggles with studies or exams as well as the mentor's own mentoring experience have a positive influence on the student's evaluation. This factors most likely, give hints to the notion of approachability, openness, and similitude, all factors that contribute to the perception of likability [16]. In line with this, we also interpret the positive assessment of residents as “near-peers”. Similarly, Straus et al. described a “personal connection” and “shared values” as aspects of a successful mentoring relationship [18]. These results are in line with findings in previous interview studies [19, 20]. We therefore hypothesize that the here presented positive corelated subcategories of profile texts contribute to the perception of likeability and therefore play a relevant role in the choice of a potential mentor. This could provide possible explanations for the results of a survey looking at selection criteria by means of an online matching process, where “likeability” based on the online profile text composed by the mentor was the most mentioned reason for selection [13]. Assessment of likeability in online matching formats, might be a challenge and probably explains why matching strategies that involve a personal encounter are often preferred by mentoring program coordinators.

Third, our results (on *hot topics*) emphasize the importance of predefined goals by the students when entering a mentoring relationship [13]. This underlines that in addition to formal categories and likeability, content-related mentoring aspects also have a significant impact on the profile evaluation and therefore selection of mentor.

Due to globalization and improved technical possibilities, web-based mentoring is a growing field [21, 22]. The recent pandemic has aggravated this trend [23–25]. Selection of a suitable mentor online is challenging, since in the perception of future mentees lack of personal encounters make assessment more difficult. Therefore, various approaches have succeeded in developing new medical mentoring databases that mentees can use to select their mentor [26–28]. However, to the best of our knowledge, literature on analysis of EDP-supported matching processes and profile text analysis in academic mentoring is limited. Similar matching processes have previously been explored in online health communities where patients aim to identify suitable peer mentors [29, 30]. Here, usage of language and selection of vocabulary similarity was found to be more important than the influence of demographics and information on health interests in selection of mentors [29, 30]. This was interpreted as prioritization for interpersonal compatibility. Moreover, personal similarities have been described in existing mentoring dyads as the basis for successful mentoring [31].

Limitations arise from the fact that only the free texts of mentoring profiles were analysed and not the whole profile. However, mentoring profiles in our context were only composed by name, specialization, picture, and text, with text providing the most important information. Moreover, aim of this study was to separately look at elements in profile texts that inform students´ decision. The introduced categorization system has been validated internally based on profile texts of our own mentoring program. External validation is pending. Though, most parts of the here presented categorization is not specific to our program or even medicine, therefore we believe that most results could inform directors of different mentoring programs. Another limitation poses the fact, that we were not able to compare profile texts based on real life data on selection of mentors. Due to differences in number of mentees each mentor cared for, duration of those relationships and number of students interested in the different medical specialisations this kind of analysis was not possible. To what extent motivation and diligence in the creation of the profile texts and the quality of mentoring relationships is related must be assessed in follow-up studies. An additional limitation arises from personal preferences of the students for certain mentor characteristics, which might impact the evaluation behavior of the profile texts.

To our knowledge this study is the first to analyse mentoring profile texts in the medical, academic environment. Strengths of the study is the high number of profile texts analysed, as well as the multi-method approach, including qualitative and quantitative assessment methods. We further present a categorization system for profile texts with high interrater reliability.

Based on our data we derive the following recommendations: i) Mentors should be made aware of formal criteria such as the formulation of a continuous text, usage of a salutation or impact of soft factors discussed above when creating a profile. ii) Addressing at least three *hot topics* or in other programs corresponding important topics in predefined fields could become mandatory.

## Conclusions

Finding the right mentor in an academic environment is a challenge for medical students, especially when using online mentoring databases. We have therefore defined categories that positively or negatively influence the likeability of a mentor based on his composed profile text. Formal text criteria and the addressing of known important topics to mentees in a future mentoring relationship have a significant influence. Other influencing factors in students' perception of mentoring profiles need to be examined. Although data on the influence of mentoring profile text quality on the quality of the mentoring relationship are pending, we believe that the here mentioned factors could significantly improve online matching processes.

### Supplementary Information

**Additional file 1:** **Supplement Table 1.** Characteristics of mentors related to the randomly selected mentoring profiles. Information was collected based on the profile data where available.** Supplement Table 2.** Full category-system for mentoring profile text analysis including 5 main categories, 21 categories and 74 subcategories.** Supplement Table 3.** Statistical analysis of results presented in Figure 2 and Figure 3.

## Data Availability

The datasets generated and/or analyzed during the current study are not publicly available but are available from the corresponding author on reasonable request.
