# Supplementary material for: Characteristics and quality assessment of online mentoring profile texts in academic medical mentoring
Source: BMC Med Educ. 2023 Nov 9;23:849. doi: 10.1186/s12909-023-04804-1 (PMC10636985; doi:10.1186/s12909-023-04804-1)
Supplement: Supplementary file 1 — Additional file 1: Supplement Table 1. Characteristics of mentors related to the randomly selected mentoring profiles. Information was collected based on the profile data where available. Supplement Table 2. Full category-system for mentoring profile text analysis including 5 main categories, 21 categories and 74 subcategories. Supplement Table 3. Statistical analysis of results presented in Figure 2 and Figure 3. [file 12909_2023_4804_MOESM1_ESM.docx]

# Appendices

| **Characteristics of mentors** | | |
| --- | --- | --- |
| Gender | Female  Male | 28%  72% |
| Place of study | LMU  Non-LMU  Unknown | 53%  18%  29% |
| Year of graduation | Median | 2006 |
| Current working environment | University hospital  Hospital (other than university)  Doctor´s office | 88%  8%  3% |
| Specialization | Internal Medicine  Pediatrics  Surgery  Neurology  Anesthesiology | 18%  13%  12%  8%  8% |

# *Supplement Table 1: Characteristics of mentors related to the randomly selected mentoring profiles. Information was collected based on the profile data where available.*

| **Main thematic category** | **Category** | **Subcategory** |
| --- | --- | --- |
| formal | title | professor |
|  |  | PhD |
|  |  | no title |
|  | additional designation | one present |
|  |  | multiple present |
|  |  | none |
|  | text structure | bullet points |
|  |  | formulated text |
|  | greeting | formal |
|  |  | informal |
|  |  | no greeting |
|  | pronominal form of address | on first name terms |
|  |  | usage of the formal term of address |
|  |  | no salutation |
|  | references | internet link |
|  |  | curriculum vitae |
|  |  | publications |
|  |  | other not personally written texts |
| studies | place of study | LMU Munich |
|  |  | TUM Munich |
|  |  | Germany (other than Munich) |
|  |  | abroad |
|  | change of place of study | yes |
|  | own doctoral thesis | experimental |
|  |  | clinical |
|  |  | statistic |
|  | extracurricular engagement | present |
|  | experience abroad | stay abroad |
|  |  | semester abroad |
|  |  | internship abroad |
|  |  | practical year abroad |
|  |  | internationally recognized state examination |
|  |  | other |
| work | work environment | university hospital |
|  |  | hospital (other than university) |
|  |  | doctor`s office |
|  |  | other |
| mentoring | mentoring in study | present |
|  |  | own mentoring request |
|  | expectations of future mentees | professional |
|  |  | research |
|  |  | interpersonal |
|  |  | other |
|  | offer of the mentor | network |
|  |  | doctoral thesis (experimental) |
|  |  | doctoral thesis (clinical) |
|  |  | doctoral thesis (statistical) |
|  |  | study/exam |
|  |  | scholarship |
|  |  | abroad |
|  |  | carrier |
|  |  | other |
| personal | interests | interpersonal |
|  |  | professional |
|  | hobbies | sports |
|  |  | cultural |
|  |  | other |
|  | family | married |
|  |  | children |
|  |  | other |

*Supplement Table 2: Full category-system for mentoring profile text analysis including 5 main categories, 21 categories and 74 subcategories.*

|  | *p*-value | *X*^2^ | Cramer´s *V* |
| --- | --- | --- | --- |
| **Regarding Figure 2 – Subcategories with statistically significant correlation to student ranking** | | | |
| Fully formulated text | <0,000001 | 2,74E+01 | 0,6125 |
| Informal form of address | <0,0000001 | 3,03E+01 | 0,6443 |
| To be on first-name terms | <0,0000001 | 3,09E+01 | 0,6509 |
| Resident doctor | <0,005 | 7,99E+00 | 0,3308 |
| Information on studies and exams | <0,00001 | 2,37E+01 | 0,5693 |
| Supervision of doctoral students | <0,001 | 1,43E+01 | 0,4422 |
| Sporting hobbies | <0,0001 | 1,60E+01 | 0,4675 |
| Own mentoring experience | <0,001 | 1,16E+01 | 0,3979 |
| Cultural hobbies | <0,001 | 1,19E+01 | 0,4042 |
| Additional offers | <0,01 | 9,46E+00 | 0,3599 |
| No salutation | <0,0000001 | 3,15E+01 | 0,6565 |
| No greetings | <0,00001 | 2,34E+01 | 0,5663 |
| Bullet points | <0,00001 | 2,13E+01 | 0,5340 |
| Data shown as a curriculum vitae | <0,0001 | 1,62E+01 | 0,4706 |
| **Regarding Figure 3 – Hot topics in medical mentorship** | | | |
| Mentioning 3 or more *hot topics*: high quality profiles *vs* low quality profiles | <0,005 | 7,99E+00 | 0,3308 |

*Supplement Table 3: Statistical analysis of results presented in* **Figure 2** *and* **Figure 3***.*


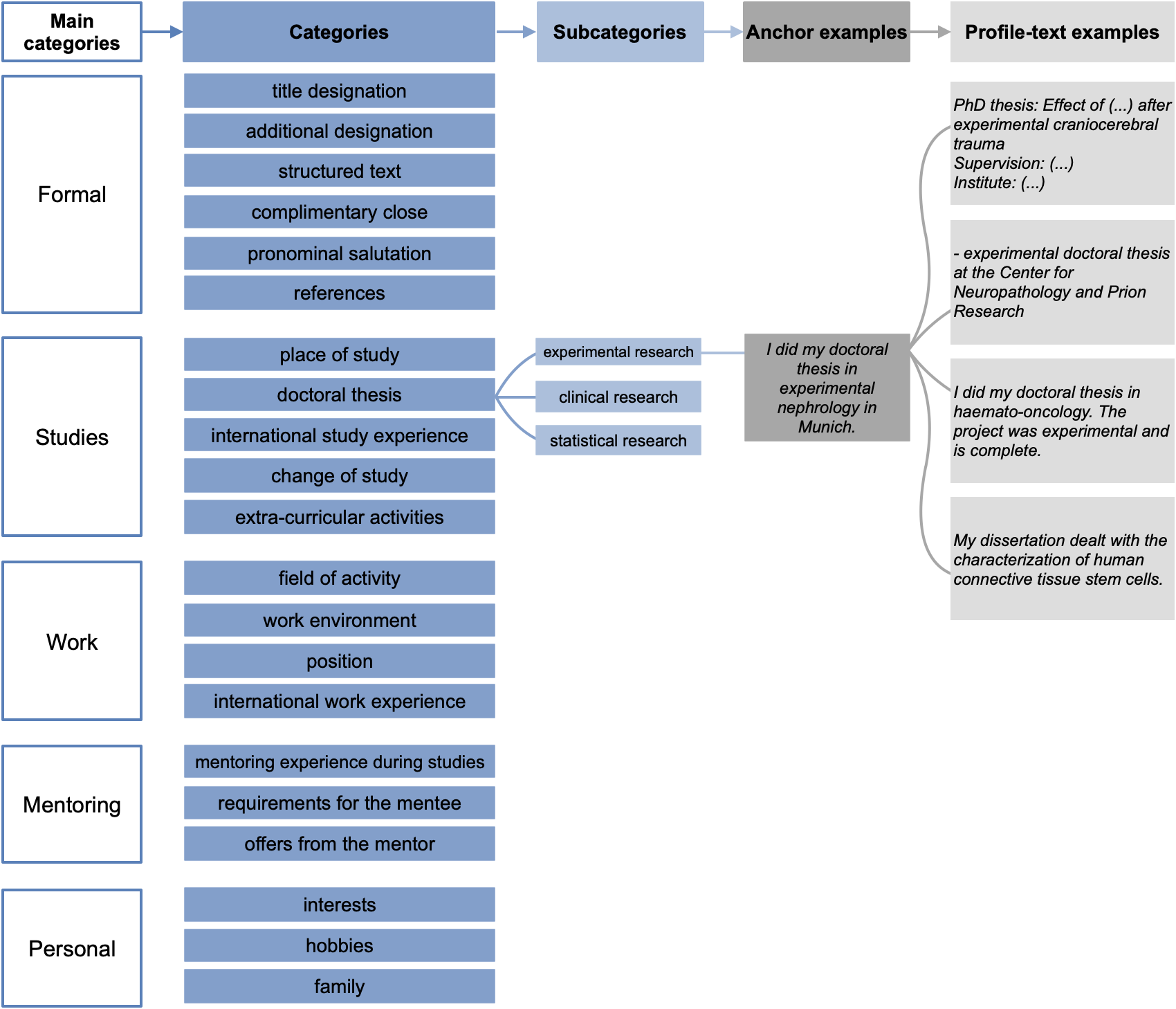


*Supplement Figure 1: Category system for qualitative analysis of mentoring-profile texts.*

*The first and second column lists the 5 main categories and 21 categories respectively. In the third column, 3 of the totals of 74 subcategories are listed as examples. An anchor example was defined for each subcategory, as shown here for the example "doctoral thesis and experimental research" (fourth column). Four exemplary quotes from the analyzed mentoring profile texts are listed in the last column.*
